# Behavioral and electrophysiological evidence for fast emergence of visual consciousness

**DOI:** 10.1093/nc/niv004

**Published:** 2015-07-30

**Authors:** Henry Railo, Antti Revonsuo, Mika Koivisto

**Affiliations:** ^1^ Department of Psychology, University of Turku, 20014, Finland;; ^2^ Centre for Cognitive Neuroscience, University of Turku, 20014, Finland;; ^3^ Brain and Mind Centre, University of Turku, 20014, Finland;; ^4^ School of Bioscience, University of Skövde, SE-54128, Sweden

**Keywords:** awareness, consciousness, theories and models, contents of consciousness

## Abstract

A fundamental unsettled dispute concerns how fast the brain generates subjective visual experiences. Both early visual cortical activation and later activity in fronto-parietal global neuronal workspace correlate with conscious vision, but resolving which of the correlates causally triggers conscious vision has proved a methodological impasse. We show that participants can report whether or not they consciously perceived a stimulus in just over 200 ms. These fast consciousness reports were extremely reliable, and did not include reflexive, unconscious responses. The neural events that causally generate conscious vision must have occurred before these behavioral reports. Analyses on single-trial neural correlates of consciousness revealed that the late cortical processing in fronto-parietal global neuronal workspace (∼300 ms) started after the fastest consciousness reports, ruling out the possibility that this late activity directly reflects the emergence of visual consciousness. The consciousness reports were preceded by a negative amplitude difference (∼160–220 ms) that spread from occipital to frontal cortex, suggesting that this correlate underlies the emergence of conscious vision.

## Introduction

Understanding the neuronal mechanisms that enable humans to consciously experience visual or any other sensory information is one of the fundamental questions of cognitive neuroscience. How long does it take for conscious visual experiences to emerge? Theories differ substantially in their answers to this question, and consequently, there are significant differences in the interpretation of empirically observed neural correlates of consciousness (NCC). A number of brain processes correlate with consciousness, but deciding which of the correlates reflect the processes that causally enable subjective experience is notoriously difficult as there is no way to directly measure subjective conscious contents, and one always has to rely on participants’ posterior subjective reports.

**Figure 1:**
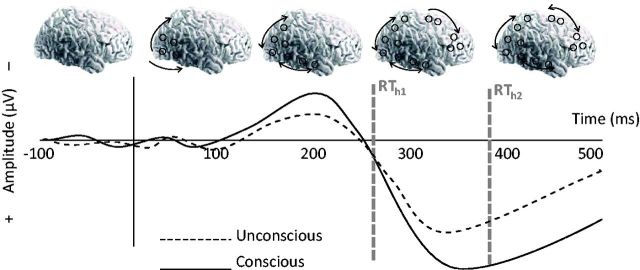
Schematic presentation of the spread of visual activation in conscious (solid line) and unconscious (dashed line) conditions (not actual data) Whereas the early vision model associates consciousness with relatively early (∼200 ms) activation within the visual cortex, the global workspace model assumes that later (after 300 ms) cortical activation in fronto-parietal cortices enables conscious vision. As shown in the ERP below, these NCCs correspond to early negative and later positive voltage enhancements in ERPs. The global workspace model predicts that the late enhancement of fronto-parietal activity must precede any reports of consciousness (RT_h2_). However, the late fronto-parietal activation cannot causally enable conscious vision if participants can report the contents of their consciousness before the fronto-parietal activation begins (RT_h1_).

Conscious vision correlates with early activation in visual cortex and with later widespread activation in fronto-parietal cortices (Lamme, 2010; Dehaene and Changeux, 2011). According to the early vision model (Lamme, 2010; Railo, Koivisto and Revonsuo, 2011), early recurrent communication of neural populations within the visual cortex enables subjective conscious perception, whereas the fronto-parietal networks are activated as conscious information is processed further and accessed by the cognitive system. The global neural workspace model (Gaillard *et al*., 2009; Dehaene and Changeux, 2011; Dehaene, 2014) posits that the early activation in visual cortex is preconscious, and can only enter consciousness if it accesses coordinated communication in the fronto-parietal global workspace. As shown in Fig. 1, in event-related potentials (ERP) that depict the brain’s electrophysiological response to sensory stimulation, the early correlate is a negative amplitude enhancement over posterior regions around 200 ms after the onset of a visual stimulus (visual awareness negativity, VAN) (Railo, Koivisto and Revonsuo, 2011). This is followed by a broad late positive (LP) enhancement around 300 ms (P300 wave), which is assumed to reflect processing in the global workspace (Gaillard *et al*., 2009). Both correlates have been suggested to reflect the neural mechanism that directly enables conscious vision, meaning that subjective visual experiences occur at the very same time as the neural correlate. 

Previous studies have not been able to conclude which of the proposed NCCs causally underlie conscious vision. Studies that have attempted to shed light on the issue have provided indirect evidence or relied on theoretical arguments, either supporting the early visual (Koivisto, Revonsuo and Lehtonen, 2006; Block, 2007; Lamme, 2010), or the global neuronal workspace model (Sergent, Baillet and Dehaene, 2005; Kouider *et al*., 2010; Dehaene and Changeux, 2011). According to the early vision model, the late NCCs are consequences of consciousness (Lamme, 2010; Aru *et al*., 2012)—they reflect cognitive processing of information that has already gained access to consciousness. In contrast, the proponents of the global neuronal workspace model (Dehaene and Changeux, 2011; Dehaene, 2014) argue that the early correlates are prerequisites for consciousness—they index preconscious neural processes that precede but do not temporally overlap with access to consciousness. According to this view, consciousness is only enabled through the late processing stages.

Here we employ a simple boundary condition to test whether an observed NCC is a consequence of consciousness: If a behavioral response to a visual stimulus is causally dependent on conscious vision, then the direct NCCs must occur before the behavioral response. Our aim, more specifically, was to examine whether the late ERP correlate of consciousness directly underlies the emergence of conscious vision (Dehaene and Changeux, 2011), or whether it reflects post-perceptual processing that emerges as a consequence of conscious vision (Block, 2007; Lamme, 2010). Our rationale is the following: by measuring the latency of the earliest reports of conscious perception it is possible to draw a definite time limit before which the direct NCC must have occurred for it to be a causal antecedent of the motor response. In our paradigm, participants were asked to respond as soon as they consciously detected a visual target (“fast consciousness report”), and to not respond when they did not consciously see the target (go/no-go task). Afterward, the participants rated how well they subjectively perceived the stimulus. Because it is well-known that behavior may be influenced by unconscious visual information (e.g. blindsight; Cowey, 2010), it is essential to confirm that the fast consciousness reports are based on conscious vision. If the fast consciousness reports are based on conscious vision, go-responses should only occur when participants report seeing a stimulus. Unconscious, blindsight-like behavior would be revealed if go-responses occur also when participants report that they did not consciously see a target stimulus. Importantly, the go/no-go task had minimal cognitive demands and only required participants to access the presence of simple visual conscious percepts as we wanted to estimate the latencies of the earliest reports of subjective vision. Go/no-go tasks have previously been used to examine the speed of processing in the visual system, for example, by asking participants to categorize natural images (Thorpe, Fize and Marlot, 1996; Li *et al*., 2002). A crucial difference from these studies is that in the present study participants were specifically asked to access their conscious visual contents.

Transcranial magnetic stimulation (TMS) of early visual cortex disrupts conscious vision of simple stimulus features most reliably about 90–100 ms after the stimulus onset, whereas at later latencies (> 200 ms) performance in tasks requiring more complex processes (e.g. visual search or binding) may be impaired (de Graaf *et al*., 2014). By employing TMS of primary visual cortex (V1) at 90 ms, we were able to create conditions in which a high contrast visual stimulus was sometimes very clearly consciously visible and sometimes not at all consciously visible, while keeping the physical stimulation identical across conditions. Although V1 TMS per se can be argued to interfere with preconscious vision, the NCCs (VAN and LP) should still be observed in the later stages of visual processing. Using concurrent electroencephalography (EEG) measurement we were able to compare the latencies of the reports of consciousness to the NCCs in the same trials within participants. In previous studies the NCC has been calculated across participants by averaging over single-trials, thus losing the information available at single-trials. The results show that the late enhancement of neural activation (LP) associated with widespread activation of fronto-parietal cortices and conscious access (Dehaene and Changeux, 2011), starts after fast consciousness reports of consciousness, and it is thus too late to causally enable conscious vision.

## Materials and Methods

### Participants

Seven neurologically healthy right-handed participants (aged 22–34 years, 3 males, including author H.R.) with normal or corrected-to-normal vision took part in the experiment, and were paid 20€/hour for participation. Informed written consent was obtained before the experiment. The study was conducted in accordance with the Declaration of Helsinki, and it was approved by the ethics committee of Hospital District of Southwest Finland.

### Stimuli and Procedure

We asked participants to respond as soon as they consciously perceived a target stimulus in the left or right hemifield (go/no-go task). Visibility of the target was manipulated by left or right visual cortex TMS, delivered 90 ms after visual stimulus onset. Contralateral TMS (with respect to visual stimulus) was used to suppress the visibility of visual targets, while the ipsilateral condition served as a control condition (Amassian *et al*., 1989). Thus, the contralateral condition served as the suppressed consciousness (or unconscious) condition and the ipsilateral condition served as the conscious condition. We employed the 90 ms TMS delay to maximize the suppressive effect of TMS. Hence our aim is not to target VAN or LP specifically with TMS, but to produce a condition in which conscious visibility is decreased (contralateral) or not (ipsilateral).

Visual stimuli were presented on a gray background (8.3 cd/m^2^) on a 19-inch CRT monitor set to 85 Hz (11.8 ms/frame). The target stimulus was a 0.8° black and white Gaussian luminance-modulated grating (5 cycles/degree) which was presented in the lower left or right hemifield (2.1° from fixation) for one screen refresh. A fixation cross was presented in the middle of the screen before (800–1700 ms), during, and after (1500 ms) the target stimulus.

The participant’s task was to fixate the center of the screen, and press a button on a gamepad as soon as she saw a stimulus in the lower left or right quadrant of the screen. They were instructed not to press the button when they did not see the target stimulus. Depending on the test session, this go/no-go response was given using the index finger of the left or right hand (order was counterbalanced). After the go/no-go response the participant reported what she had seen by pressing one of four buttons on the gamepad with her right thumb (Sandberg *et al*., 2010). The response alternatives were: 0—did not see anything, 1—I saw a brief glimpse of the target, 2—I saw the target but it was somewhat unclear, or 3—I saw the target clearly.

Each participant participated in two test sessions performed on separate days. During each session a participant completed 5–7 experimental blocks consisting of 56 trials. Each block contained the following conditions, presented in random order: 1—Visual target presented alone (Vis_only_), 2—Visual target presented together with TMS (contralateral or ipsilateral with respect to visual target), and 3—TMS applied without a visual target (TMS_only_). During the experiment, contralateral, ipsilateral and Vis_only_ trials were each presented 192 times, and TMS_only_ trials were presented 96 times for each participant.

### EEG recording and preprocessing

EEG was recorded continuously at 20 kHz in DC mode with 32 Ag/AgCl electrodes using the NeurOne Tesla amplifier (Mega Electronics). Thirty electrodes were placed on the scalp based on the International 10–20 System. One electrode was placed below the right eye and another at the right outer canthus. Reference electrode was placed on nose and ground electrode on forehead. Electrode impedances were brought below 5 kΩ in the beginning of the recording.

EEG was processed using EEGLAB (Delorme and Makeig, 2004) (versions 11 and 13) and Matlab (version 7.9.0). Before preprocessing, EEG was downsampled to 5 kHz, and the TMS pulse artifact was removed by cutting out 15 ms of EEG (beginning three samples before the pulse). This “mute window” was then interpolated by a third-order polynomial curve (Supplementary Fig. 1a; see Reichenbach, Whittingstall and Thielscher, 2011, for a similar procedure), and the signal was resampled to 250 Hz. To minimize the temporal smearing of the ERP waves due to filtering, the data was first high-pass (0.05 Hz half-amplitude cutoff) and then low-pass (40 Hz half-amplitude cutoff) filtered using a one-pass Blackman windowed sinc filter (VanRullen, 2011; Widmann and Schröger, 2012, Widmann *et al*., 2015). The EEG was segmented to -200–500 ms epochs, and clearly artifactual epochs were removed based on visual inspection. Next, an independent component analysis (ICA; runica algorithm) was run, and epochs that contained improbable data were removed based on the ICA (activity threshold in single components: 5 SD; 16% of all epochs removed in total). A second ICA was run to remove eye movement (Jung *et al*., 2000) and strong TMS-related artifact components (Hamidi *et al*., 2010; Korhonen *et al*., 2011) before ERP analysis. If a component appeared to contain some visual stimulus-related effects, it was not removed from the data. Removed TMS-related artifact components localized to occipital electrodes, and only contained one sharp peak right after the pulse (see Supplementary Fig. 1b).

### Transcranial magnetic stimulation (TMS)

A Nexstim eXimia (Helsinki, Finland) stimulator was used for TMS. A focal biphasic 70-mm figure-of-eight coil was fixed to a tripod during stimulation, and current direction was from lateral to medial during second phase of the pulse. TMS intensity was 70% of the stimulator’s maximal output. The stimulator’s capacitors were recharged 1 s after the pulse to avoid EEG artifacts.

The position of the coil was continuously registered relative to the participants’ anatomical brain image using eXimia Navigated Brain Stimulation system. Anatomical images were acquired with magnetic resonance imaging using a high-resolution T1-weighted 3D-sequence. Single TMS-pulses were applied to the upper bank of the participant’s calcarine sulcus as it accurately predicts the location of an individual’s V1 (Hinds *et al*., 2008). Left and right hemispheres were stimulated by turns in different blocks (order was counterbalanced).

### Statistical analysis

The behavioral data was analyzed in SPSS 19 using a repeated measures TMS (2: left, right hemisphere) × Target (2: left, right hemifield) analysis of variance (ANOVA). The *P*-values were Greenhouse-Geisser corrected when the sphericity assumption was not met. Reaction time (RT) analyses are based on median RTs of correct go-responses. To increase statistical power, the relationship between RTs and visibility ratings in the contralateral TMS condition was assessed using a linear mixed-effects model (Bayeen, Davidson, and Bates, 2008) in *R* statistical software (R Development Core Team, 2014). In the model, visibility rating was defined as a fixed factor, and participants were defined as random-effects factors with individual intercepts. The model was fit (maximized log-likelihood) using the nlme package (Pinhero *et al*., 2014). Because the RT distributions were skewed to the right, all RT analyses were performed on log-transformed data. In the “Results” Section we report untransformed data to make the interpretation of the results easier.

Standard grand-average ERPs were analyzed with the Mass Univariate Toolbox (Groppe *et al*., 2011) using two-tailed repeated measures *t*-tests at all 32 electrodes. The comparisons were performed on mean activity in time-windows that corresponded to the previously reported VAN, and LP/P300 time-windows (Railo, Koivisto and Revonsuo, 2011). In VAN, which revealed a “peaky” waveform profile (Fig. 4), statistical analysis was based on a 12 ms time-window around the peak (166 ms for posterior VAN, and 216 ms for frontal VAN). For the broader LP (Fig. 4), mean amplitudes were calculated based on 300–400 ms. The Benjamini and Yekutieli (2001) multiple comparison correction procedure was employed to assess the significance of each test using a false discovery rate level of 5% despite the fact that tests were performed at each electrode.

In addition to analyzing the standard grand-average ERPs and their differences we calculated the ipsilateral–contralateral difference based on single-trials to examine how the timing of VAN–LP combination related to RTs in single-trials, and within participants. The single-trial difference waves were calculated for each participant by first equating the number of trials in ipsilateral and contralateral conditions (random trials were removed from the condition that contained a higher number of trials). Then the ipsilateral and contralateral trials were sorted according to the amplitude of the most prominent TMS-evoked potential P45 (so that contralateral trials with strong P45 amplitude will be subtracted from ipsilateral trials with strong P45 amplitudes; see Fig. 6). ERP-images of ipsilateral and contralateral conditions (including RTs in the ipsilateral condition) were smoothed using five trials-wide moving averages to remove high-frequency noise. Contralateral trials were then subtracted form ipsilateral trials, and the resulting ERP-image was sorted according to (smoothed) RTs in the ipsilateral condition. Finally, the difference data was smoothed (Gaussian smoothing, SD = 2 trials) for better visualization. For a somewhat similar procedure using single-trial differences, see Bishop and Hardiman (2010).

To increase the signal-to-noise ratio of the single-trial data, each participant’s difference data was de-noised using an automated wavelet-based method (Ahmadi and Quian Quiroga, 2013). Briefly, the process is the following: in the first stage, the participant’s average ERP is decomposed using a wavelet transformation defined at different frequency scales (in the present study, four scales were used). Second, the wavelet coefficients that characterize the average evoked-response are selected by a thresholding procedure. Finally, these coefficients are applied to the single-trial data to separate the evoked responses from the background EEG activity. For details concerning the de-noising procedure, see Ahmadi and Quian Quiroga (2013).

For analyses on single-trial differences, the significance of the resulting ipsilateral–contralateral difference wave was assessed using permutations tests with a strict two-tailed significance threshold (*P* = 0.001) using EEGLAB. This analysis was performed on 1 frontal (Fz) and 1 occipito-parietal channel (Pz).

Onset latencies of VAN were estimated from the de-noised single-trials by searching for the earliest sample where amplitude was smaller than -1.5 µV for at least 20 ms after visual stimulus onset. Similarly, for estimating LP-onset latencies in de-noised single-trial differences, the criterion was: the earliest sample where amplitude is larger than 1.5 µV for at least 20 ms after VAN onset (or after stimulus onset, if the amplitude never crossed the -1.5 µV VAN threshold). We also experimented with more lenient or strict onset criteria, but they did not significantly change the results.

### Data availability

Data are not publically available but the authors will consider reasonable requests for access, for purposes of verification.

## Results

### Behavioral results

**Figure 2:**
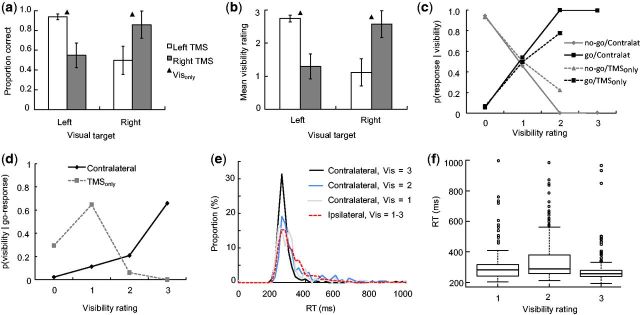
Behavioral results **(a)** Go/no-go accuracy, and **(b)** subjective visibility was decreased for contralateral relative to ipsilateral targets due to early visual cortex TMS (*N* = 7). The triangles depict the Vis_only_ condition. Error bars show the standard error of the mean. **(c)** The probability of making a go or no-go response given certain visibility rating, p(response|visibility rating), separately for contralateral (solid lines) and TMS_only_ (dashed lines) conditions. **(d)** The probability of reporting a certain visibility rating, given that the participants made a go-response, p(visibility rating|go-response), in contralateral (solid black line) and TMS_only_ (dashed gray line) conditions. **(e)** RT distributions (20 ms bin size) of correct go-responses in the ipsilateral and contralateral TMS conditions. For the contralateral condition (solid lines), separate RT distributions are shown for different visibility ratings (Vis). **(f)** Boxplots showing RT as a function of visibility rating in the contralateral condition. Figures (c)–(f) do not show the data for different visibility ratings in the ipsilateral condition because in the ipsilateral condition the participants typically chose the highest visibility rating.

As shown in Fig. 2a and b, contralateral TMS strongly decreased go/no-go response accuracy (TMS side × Target side: F_1, 6_ = 15.3, *P* = 0.008, $\eta_{\text{p}}^{2}$ = 0.72), and subjective visibility ratings (TMS side × Target side: F_1, 6_ = 22.0, *P* = 0.003, $\eta_{\text{p}}^{2}$ = 0.78). Figure 2c shows that the probability of making a go-response increased as visibility of the stimulus increased, and vice versa. When no visual stimulus was presented (i.e. TMS_only_ condition) the participants correctly did not make a go-response in 83% (SD = 16%) of trials on average (range 6–48 trials per participant). Figure 2d shows the probability of different visibility ratings, given that the participants made a go-response, separately for the contralateral and TMS_only_ conditions. If the participants relied on conscious vision in performing the go/no-go task, go-responses should occur only when participants report seeing the target. Indeed, in the contralateral condition, when the participants pressed the go-response, they reported that they did not see the target (lowest visibility rating) only on 3% (SD = 5%) of the cases (Fig. 2d). However, when no target was presented, and the participants reported not seeing the target at all (lowest visibility rating), they pressed the go-response on 5% of trials (SD = 10%; Fig. 2c). In sum, the go-responses accurately reflect the participants’ conscious vision. This is also revealed in the strong correlation between go/no-go accuracy and visibility ratings (r = 0.91, *P* = 0.005, *N* = 7). 

The median RT to consciously seen (three highest visibility ratings) ipsilateral targets was 301 ms (SD = 44.6 ms) and 269 ms (SD = 98.2 ms) to consciously seen (three highest visibility ratings) contralateral targets, showing that participants could reliably report their conscious vision remarkably fast. As shown in Fig. 2e, the fastest reports of consciously seen targets only took a little over 200 ms. There were not enough trials to reliably plot the distribution of RTs when the participants pressed the go-response, but reported not seeing the target (a total of 28 trials in the contralateral condition; median RT = 280 ms, SD = 147.3 ms).

RTs were not statistically significantly modulated by TMS (TMS side × Target side ANOVA: interaction *P* = 0.58, main effect *p*s ≥ 0.15). To see if RTs were modulated by target visibility in the contralateral condition, we performed a linear mixed-effects regression on single-trial data. The results revealed a statistically significant effect, showing that RTs decreased as visibility increased (*β* = −0.06, df = 669, *t* = −3.27, *P* = 0.001), but as seen from Fig. 2f, the absolute size of this effect is small. Importantly, however, the highest visibility rating revealed the fastest RTs. Similar test could not be performed in the ipsilateral condition, because the participants typically chose the highest visibility rating.

### TMS–EEG results

**Figure 3:**
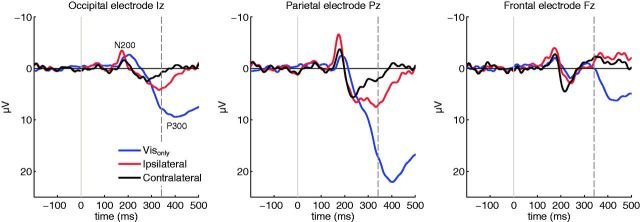
Visual ERPs in Vis_only_, ipsilateral (conscious), and contralateral (suppressed consciousness) conditions time-locked to the presentation of the visual stimulus The TMS-evoked potential activity has been removed from the ipsilateral and contralateral conditions. The vertical dashed line shows the median RT in Vis_only_ condition.

To simplify the presentation of the results, EEG data was pooled across left and right hemisphere TMS conditions. This allows a straightforward comparison of conditions where a visual stimulus was presented ipsilaterally or contralaterally with respect to TMS. The left and right hemisphere TMS conditions were also analyzed separately and none of the NCC effects showed lateralization to left or right hemispheres. To examine visual ERPs without the artifact produced by TMS, the TMS-evoked potential (TMS_only_ condition) was subtracted from the potential evoked by the ipsilateral and contralateral conditions. The resulting visual ERPs were clear with prominent N200 and P300 waves in all conditions (Fig. 3). When compared to the Vis_only_ condition, the ipsilateral and contralateral conditions (i.e. with TMS) produced somewhat decreased N200 latencies, and decreased P300 amplitudes. These effects could in part be due to nonlinear interaction effects between TMS and visual evoked potentials (Reichenbach, Whittingstall and Thielscher, 2011). The TMS conditions were also behaviorally more demanding than the Vis_only_ condition, not only because TMS could disturb task-related processing (especially during contralateral trials), but because the participants had to restrain themselves from responding to the TMS pulses alone. This may explain the decrease in P300 amplitude as it is known to vary with task difficulty and subjective confidence (Donchin and Coles, 1988; Eimer and Mazza, 2005). However, note that the crucial comparison in the present study is between the ipsilateral and contralateral TMS conditions which are physically identical, but different with respect to subjective visual experiences. 

**Figure 4:**
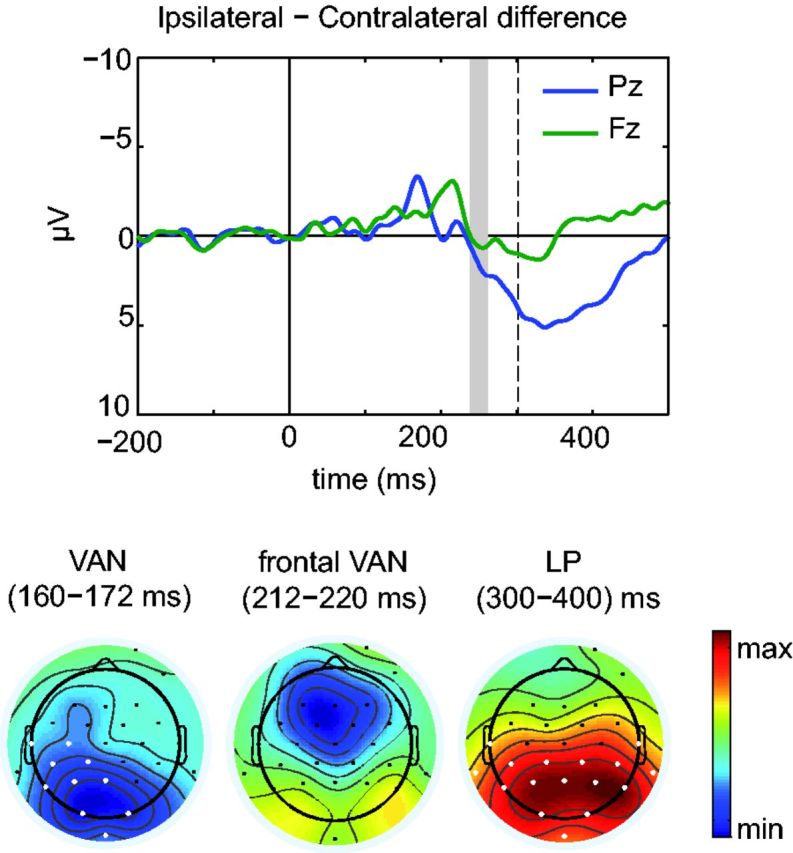
Grand-average ipsilateral–contralateral visual ERP difference reveals the early negativity (VAN) and LP as the NCCs The dashed line indicates median RT in ipsilateral condition, and the gray shaded area represents the estimated (average) latency when the command to make the go-response was initiated in the brain (40–60 ms before the actual movement). In the scalp distributions the data has been restructured so that the left hemisphere is always the stimulated hemisphere.

**Figure 5:**
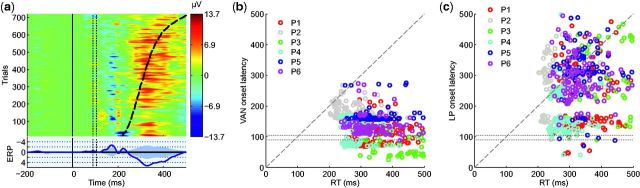
Single-trial correlates of consciousness **(a)** ERP-image of the de-noised single-trial difference data (six participants) from parietal channel (Pz) sorted by RT (dashed line). Each color-coded horizontal line depicts a single-trial ipsilateral–contralateral difference: the blue area before 200 ms is the VAN, and the red area (overlapping with RT) the LP. This grand-average ERP image has been further smoothed for better visualization (Gaussian smoothing, SD = 4 trials). In the average difference ERP presented in the lower part of the figure, the shaded area represents two-tailed alpha level 0.001 (permutation test). The vertical dotted lines show the time-period of the removed TMS-pulse artifact. **(b)** VAN and **(c)** LP onset latencies (0 ms = visual stimulus onset) in single-trials as a function of RT. Different colored symbols depict different participants. The horizontal dotted lines show the time-period of the removed TMS-pulse artifact.

One participant whose behavioral results did not show any visual suppression by TMS (ipsilateral–contralateral performance difference in accuracy = 0.1, and in visibility ratings = 0.01) was excluded from EEG analyses, as our aim was to specifically study the correlates of consciousness. First, we calculated the NCC difference wave by subtracting ERPs in the contralateral (unconscious) condition from those in the ipsilateral (conscious) condition. As shown in Fig. 4, this traditional grand-average ERP difference wave revealed the often reported NCC (Railo, Koivisto and Revonsuo, 2011): Compared to contralateral stimuli, ipsilateral stimuli first produced a negative amplitude enhancement over occipital sites (VAN), which was followed by a broad positive enhancement (LP). Statistically significant differences between contra- and ipsilateral conditions, corrected for multiple comparisons, are indicated by white electrodes in Fig. 4.

**Figure 6:**
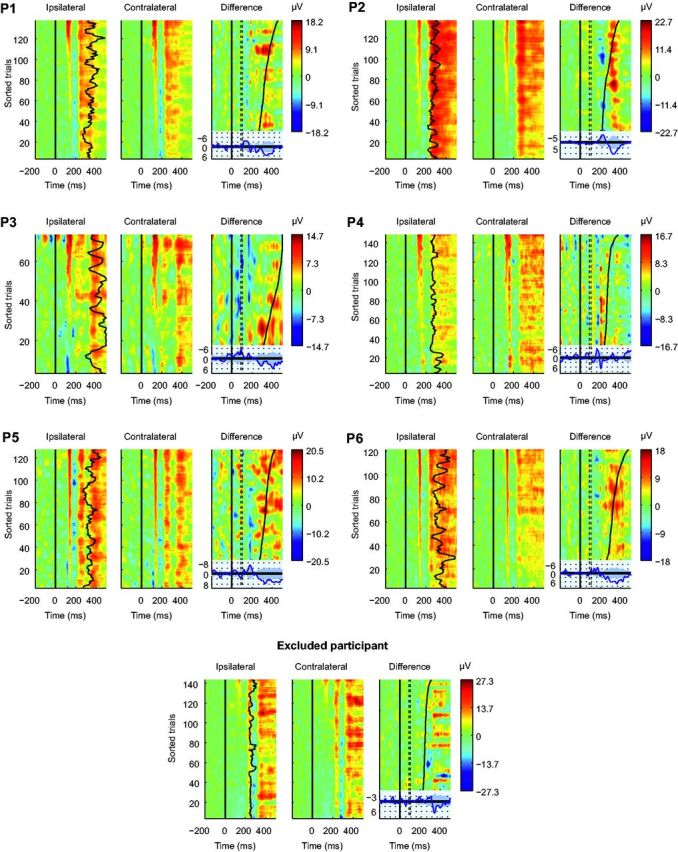
Each participant’s single-trial ERP images (channel Pz) In each panel (P1–P6, and Excluded participant), the two first ERP images show ipsilateral and contralateral trials (with the TMS artifacts remaining) sorted according to the TMS-evoked potential P45 amplitudes (vertical red area around 135 ms after visual stimulus onset). The rightmost ERP-image in each panel shows the ipsilateral–contralateral difference (i.e. NCC) with trials sorted according to RTs. The color bar shows the amplitudes of the ipsilateral–contralateral difference. The dotted lines show the time-period of the removed TMS-pulse artifact.

To examine how the NCCs relate to the participants’ fast consciousness report RTs, the ipsilateral–contralateral difference wave was calculated based on single-trials (for details see Section “Statistical analysis”; Fig. 6). Note that the RTs overestimate the timing of conscious vision as they include the time related to the execution of movements (e.g. neuromuscular transmission and the physical movement). We have *not* subtracted these “additional” time costs from the RTs. To more directly compare the latencies of the NCCs to the estimated latency of conscious access one needs to subtract approximately 40 ms from the RTs to take into account the duration of executing the button-press response (Makeig *et al*., 2004). The dashed line represents the median RT in the ipsilateral condition, whereas the estimated latency when the command to make the go-response was initiated in the brain is depicted by the gray shaded area in Fig. 4. 

The comparison of VAN and LP to RTs at the level of single-trials is shown in Fig. 5a. As seen from the minimal baseline noise in single-trial data (Fig. 5a), the de-noising successfully removed EEG background noise, but kept the single-trial NCCs (Supplementary Fig. 2 shows the data without the de-noising). Visual inspection of Fig. 5a suggests that the early negative amplitude enhancement (VAN) preceded the RTs, whereas the LP enhancement began after, or overlapped with the RTs. As shown in Fig. 6, individual participants’ data revealed similar results. Figure 6 also shows that the participant who was excluded from group analyses because she showed no suppression of visual consciousness revealed the smallest VAN (peak amplitude = −2.7 µV, barely crossing statistical significance), but rather large LP (peak amplitude > 6 µV). Consistent with our other findings, this suggests that the LP does not reflect conscious visual experiences.

To quantitatively describe the relationship between the NCCs and RTs, the onset latencies of VAN and LP were estimated based on the de-noised single-trial differences. The estimated onset latencies of VAN are plotted as a function of RT in Fig. 5b. The onset latencies are scattered around 150 ms, which corresponds to the visual inspection of the data in Fig. 5a. Some estimates of VAN onset latencies precede the TMS pulse, indicating that they do not directly reflect the difference between contralateral and ipsilateral TMS conditions. Because the pre-TMS time-windows were identical in the two conditions, these estimates likely reflect random EEG fluctuations which the de-noising procedure (incorrectly) identified as VAN. However, for the majority of trials, the estimates show that VAN onset latency is after the TMS pulse, but before manual RTs. The estimated onset latencies of LP, shown in Fig. 5c, reveal two clusters of data points. The cluster with the earlier onset latency is too early to reflect the LP, and it coincides with the TMS-evoked potential P45 (135 ms after stimulus onset; see Fig. 6). Comparison with Fig. 5a indicates that the second cluster of onset latency estimates corresponds to the LP. Importantly, in many cases (36.2 %) these onset latencies follow the manual RTs in this cluster.

**Figure 7:**
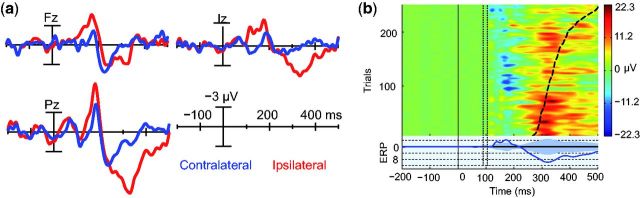
Comparison of strictly unconscious vs. conscious ERPs **(a)** ERPs in ipsilateral and contralateral conditions with only conscious (ipsilateral; two highest visibility ratings; red lines) versus unconscious (contralateral; lowest visibility rating; blue lines) trials included. In the mass univariate statistical analysis, only VAN reached significance (*N* = 4). **(b)** De-noised single-trial differences of conscious ipsilateral and unconscious contralateral trials (*N* = 4), sorted according to RTs (dashed line), from the electrode Pz. In the average difference ERP presented in the lower part of the figure, both VAN and LP cross-statistical significance (permutation test, shaded area represents two-tailed alpha level 0.001). The dotted vertical lines show the time-period of the removed TMS-pulse artifact.

Similar correlates of consciousness were observed when the analysis included only unconscious contralateral trials (lowest visibility rating) and only conscious ipsilateral trials (two highest visibility ratings). Due to insufficient amount of trials with complete visual suppression, this comparison (shown in Fig. 7a) is only based on four participants. Although statistical power is thus limited, the results closely resemble the ipsilateral versus contralateral condition, and verify that the observed ERP differences are correlates of consciousness. Furthermore, single-trial difference waves show that whereas VAN always preceded the RTs, LP overlapped with, and in the fastest RTs began after, the RTs (Fig. 7b). 

VAN peaked later in the frontal than in the parietal recording sites. The frontal VAN is visible in the conventional grand-average analysis (Fig. 4a), although it only reached statistical significance in the single-trial differences (electrode Fz, *N* = 6, *P* < 0.001, two-tailed permutation test). Overall, the single-trial differences reveal very similar results as the conventional grand-average differences (compare the shapes of the blue ERP lines in Figs 4 and 5a), but allow the examination of the dynamics of the NCC within single participants.

## Discussion

Our results demonstrate that participants can accurately report the presence of simple conscious visual contents extremely fast, in little over 200 ms. The onset of the consciousness-related enhancement of the P300 potential (LP), which has been argued to enable the access of conscious contents, begins after or overlaps with these fast reports of conscious perception. Because neuromuscular conduction, direct motor commands (Makeig *et al*., 2004), and decision-making processes (de Lange *et al*., 2012) are included in the RTs, they overestimate the latency of conscious access. Thus the LP is too late to reflect the neuronal processes that causally enable subjective visual experiences to guide behavior. The negative amplitude consciousness-related enhancement (VAN), which started after 100 ms and peaked prior 200 ms is more likely to reflect the neural processes that causally enable conscious vision as it always preceded manual responses. The early part of VAN is posterior, suggesting visual cortical origin. Extending this previously reported finding (Koivisto and Revonsuo, 2010; Railo, Koivisto and Revonsuo, 2011), we show that VAN later shifts to frontal regions. Our conclusions about the timing of the causal mechanisms generating consciousness rest on the assumption that the participants based their go-responses on conscious vision. The fact that we manipulated consciousness with TMS, which allows studying whether specific cortical areas causally contribute to cognitive functions, is irrelevant for the present conclusions.

During the VAN time-window (150–200 ms), intracranial recordings in ventral visual cortex show enhanced and sustained activation (Fisch *et al*., 2009) and gamma oscillations synchronize across cortex (Melloni *et al*., 2007) during conscious vision. We suggest that the early posterior VAN reflects enhanced neuronal processing that allows visual stimuli to cross the threshold to sensory consciousness (Fisch *et al*., 2009; Lamme, 2010; Railo, Koivisto and Revonsuo, 2011). This enhanced neural activity in visual cortex may then enable communication with more distant regions, such as frontal cortices, allowing phenomenally conscious contents to guide visuomotor behavior, as reflected in the frontal VAN (Lamme, 2010). The fact that participants could consciously access visual contents in just over 200 ms in the present study suggests that conscious perception of object presence may be enabled by fast local recurrent neural interactions (Lamme, 2010), or even stimulus-driven feedforward activation (Bar, 2003; Fabre-Thorpe, 2011; Campana and Tallon-Baudry, 2013). The function of later recurrent processing could be to clarify and increase the resolution of conscious vision (Hochstein and Ahissar, 2002; Campana and Tallon-Baudry, 2013). Consistent with this, the early part of VAN (< 200 ms) has been shown to emerge independent of top-down feature-based attention, although the two interact at later stages of processing (Koivisto, Revonsuo and Lehtonen, 2006; for reviews see, Railo, Koivisto and Revonsuo, 2011; Koivisto and Revonsuo, 2010). Spatial attention, however, may be a prerequisite of conscious vision (Koivisto, Kainulainen and Revonsuo, 2009), and in the present study, we cannot rule out the possibility that VAN was also modulated by spatial attentional selection.

Early ERP differences are also observed when participants categorize natural scenes using fast go/no-go responses (Thorpe, Fize and Marlot, 1996). However, the natural scene categorization RTs are clearly longer (median RTs > 400 ms; Thorpe, Fize and Marlot, 1996; Li *et al*., 2002; Koivisto, Kastrati and Revonsuo, 2014) than the fast consciousness reports observed in the present study. Whereas the fastest rapid visual categorization responses have been assumed to be based on unconscious visual information (Fabre-Thorpe, 2011), our results strongly suggest that participants can consciously access visual information in just over 200 ms. In line with our results, Koivisto *et al*. (2014) observed that participants typically reported being conscious of the scene contents during the fastest scene categorization responses, and observed that RTs increased as the rated level of consciousness decreased (Koivisto *et al*., 2014). In the study, scene visibility was manipulated by object substitution masking, which is assumed to interfere with recurrent processing (Di Lollo, Enns and Rensink, 2000). As object substitution masking did not decrease categorization accuracy, but slowed down RTs, the authors concluded that whereas conscious natural scene categorization may rely on fast feedforward inputs, recurrent processing was necessary for detailed conscious perception (Koivisto, Kastrati and Revonsuo, 2014). Although the present results imply that simple visuomotor access of conscious contents is independent of late processing in the global workspace, more complicated, attention-dependent manipulation (e.g. conscious recognition) of conscious visual contents could require the late activity.

The present findings are in line with earlier reports that have dissociated the LP and conscious perception. By using visual masking, Koivisto *et al*. (2006) observed that LP amplitude was strongly reduced when participants attended to local target features as compared with attending to global features, although in both conditions the participants were aware of the stimulus (See also Eimer and Mazza, 2005; Koivisto and Revonsuo, 2008). These findings were recently extended by Pitts *et al*. (2014), who showed that the P3 was only observed when participants consciously processed task-relevant information. The authors noted that the P3 could thus still be considered sufficient, but not necessary, for conscious perception. However, a P3 wave may be elicited even by stimuli that remain completely outside consciousness (in the auditory modality; Cavinato *et al*., 2012), suggesting that it can be completely dissociated from conscious perception.

As stimuli that are suppressed from consciousness by early visual cortex TMS have been shown to influence behavior (Ro *et al*., 2004; Railo and Koivisto, 2012), it can be argued that the fastest responses were based on unconscious information, and that the visual information later crossed the threshold to consciousness. However, our data does not support this conclusion, and can be more parsimoniously explained by assuming the participants were conscious of the visual stimuli when they made the go-response. First, the above-mentioned counterargument makes the assumption that when the unconscious visual information was strong enough to guide manual responses it was also strong enough to later cross the threshold to consciousness, and that when the stimulus processing was so weak that it could not be consciously perceived, it could not drive unconscious responses either. Although this possibility cannot be ruled out, we think that such a strong association between unconscious and conscious processing is implausible. Rather, it makes more sense to argue that one of the functions of consciousness is to guide behavior in situations that require fast responses to weak stimuli. Second, consistent with our interpretation, the trials with the highest visibility ratings yielded the fastest RTs. Third, the participants were instructed to only press the response button if they consciously saw the target. The go-responses strongly correlated with visibility ratings which have been shown to provide a reliable measure of conscious visual contents (Sandberg *et al*., 2010). This, and the fact that the participants rarely pressed the go-response when they reported not consciously seeing the target (i.e. they obeyed with the instructions), suggests that the go-responses reliably indicated the presence of conscious visual experiences.

Note that evidence for unconscious vision is typically gained through behavioral forced-choice accuracy measures, or through indirect RT methods such as priming (Dehaene *et al*, 1989; Stoerig and Cowey, 1989; VanRullen and Koch, 2003; Breitmeyer, Ro and Singhal, 2004; Del Cul *et al*., 2009; de Lange *et al*., 2011), whereas the task in the present study was clearly more demanding, and required direct, accurate, and fast responses to targets (that were presented among catch trials where no visual stimuli were presented). Therefore, although previous studies show that unconscious stimuli can modulate decisions (often recognition) that are triggered by a consciously perceived stimulus (e.g. a mask; Dehaene *et al*., 1998; VanRullen and Koch, 2003), we are not aware of any reports that show that participants can respond to isolated and completely unconscious visual stimuli extremely fast and with high accuracy. Moreover, although the fastest RTs are especially often deemed to be initiated by unconscious processes (which may later enter consciousness; VanRullen and Koch, 2003), this conclusion is often made on theoretical rather than empirical grounds (i.e. early coarse visual representations are assumed to be unconscious). In line with previous proposals (Hochstein and Ahissar, 2002; Campana and Tallon-Baudry, 2013), our results suggest that early visual activation (∼200–300 ms) may enable conscious access of coarse visual information (e.g. object presence), although later visual processes may be necessary for more complicated visual decisions (e.g. recognition). Note that we are not claiming that in the present study the RTs were exclusively determined by conscious processing; unconscious processes also likely influenced the RTs. However, we argue that conscious vision played a necessary causal role in triggering the behavioral responses. Nevertheless, future studies are important to verify, extend, and clarify the present findings and the influences of unconscious processing in general. For example, did the fact that we asked participants to only respond to consciously perceived stimuli affect the results (i.e. how much voluntary control do humans have on speeded judgments)? To what extent can speeded motor responses be initiated by completely unconscious information, when no other stimuli (e.g. masks) act as response cues?

Although we argue that go-responses were in the present study initiated by conscious vision, stimuli that remained completely unconscious due to TMS suppression were registered by the brain: they elicited ERP responses (Fig. 7, blue traces), but activity was significantly decreased during VAN and LP time-windows. This shows that TMS applied over the early visual cortex can block conscious vision without completely suppressing stimulus-driven activation, which may explain why stimuli whose visibility is suppressed due to early visual cortex TMS may still influence behavior (Ro *et al*., 2004; Railo and Koivisto, 2012).

Various different methods of manipulating consciousness (e.g. visual masking, attentional blink, change blindness, low-contrast stimuli, binocular rivalry) have revealed the same pattern of electrophysiological correlates of visual consciousness in which VAN is followed by LP (Koivisto and Revonsuo, 2010; Railo, Koivisto and Revonsuo, 2011). Our results show that similar ERP correlates of consciousness are observed when conscious vision is manipulated using early visual cortex TMS. The reason for using TMS as a suppression method in the present study was that it made it possible to have a conscious condition in which the stimulus was clearly visible (ipsilateral condition), resulting in fast and confident manual responses, and an unconscious condition (contralateral) in which the visibility was suppressed, while keeping both the visual stimulus and the magnetic stimulation constant. By contrast, other methods of keeping the visual stimulation identical across conscious and unconscious conditions (e.g. low-contrast stimuli or masked stimuli, presented near the threshold for consciousness) would have resulted in much slower RTs due to weak conscious perception (e.g. 725–1066 ms in Salti, Bar-Haim and Lamy, 2013). Moreover, unlike in the present study, many of the responses might have been based on guessing and weak confidence rather than on clear conscious perception.

We acknowledge that our experimental setup confounds manual responses with consciousness as go-responses were only present in conscious trials. However, we would have faced similar problems, had we, for example, employed a task that also required manual responses to the unconscious condition (also then the timing of motor-related response might overlap with the NCC). The fast go/no-go conscious reports allowed us to verify that the participants based their manual responses on conscious vision while keeping response selection simple: choice RTs would have led to longer response times. The fact that we did not observe large changes in RTs as a function stimulus visibility is consistent with the assumption that participants could base their go-responses on weak visual signals. However, the extremely low proportion of go-responses during complete visual suppression implies that the participants relied on their conscious vision in making the go/no-go decision. Had they relied on reflexive, unconscious percepts, a significant number of go-responses should have been detected on those trials where the participants reported not seeing the target.

Finally, our approach was hypothesis-driven as we expected to observe the two major candidates for the ERP correlates of visual consciousness (VAN and LP), which have both been observed in passive viewing situations that control for the potential contaminating effects of motor behavior (Koivisto and Revonsuo, 2008). Neither VAN, nor LP, revealed lateralization when analysis was restricted to right or left hand response conditions, and neither correlates with RT, suggesting that they do not directly underlie motor responding (See also McCarthy and Donchin, 1980; Makeig *et al*., 2004; Delorme, Westerfield and Makeig, 2007).

## Conclusions

The present results indicate that humans can access conscious visual contents remarkably fast and accurately, and that this does not require the activation of the fronto-parietal global neuronal workspace. At the same time, our results suggest that a widespread activation of cortical areas occurs earlier (150–200 ms) than previously assumed, supporting the early vision models of visual consciousness (Lamme, 2010). In future, the approach of relating the timing of NCC to exact timing of behavior through single-trial analysis may prove fruitful in accomplishing more detailed models of conscious access.

## Supplementary Material

Supplementary Data
