# Supplementary material for: Behavioral and electrophysiological evidence for fast emergence of visual consciousness
Source: Neurosci Conscious. 2015 Jul 30;2015(1):niv004. doi: 10.1093/nc/niv004 (PMC6368270; doi:10.1093/nc/niv004)
Supplement: Supplementary Data [file niv004_Supplementary_Data.zip › Supplementary_Figure_1-1.docx]

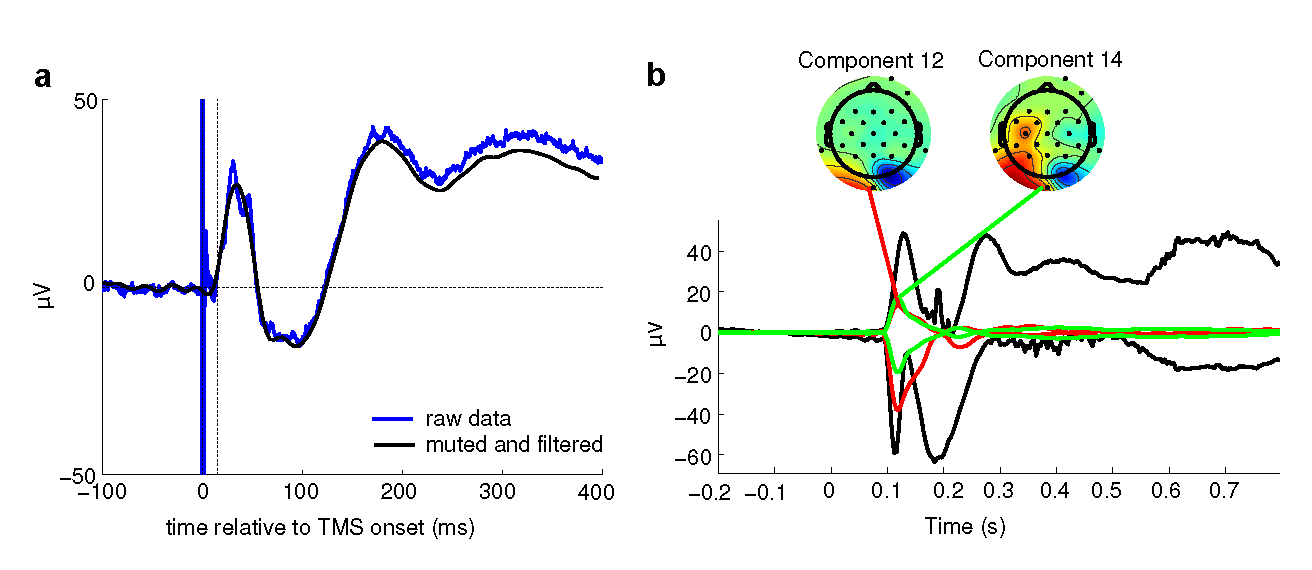


*Supplementary Figure 1. An example of TMS artifact removal and filtering. a) Averaged responses to TMS pulse in the TMS_only_ condition from electrode Pz in participant 2. The blue line shows the raw, unfiltered data with the TMS-artifact remaining and the black line shows the same data after the removal of the TMS-artifact and filtering. The vertical dotted lines show the time-window of the TMS-artifact removal. b) Examples of two TMS-related independent components from participant 2. The black traces depict the whole data envelope, i.e. the minimum and maximum voltages across all channels at all time-points. The red and green lines depict the envelopes of components 12 and 14, respectively. Scalp topographies of components 12 and 14 are also shown. The time-course of component activations and their scalp topographies indicate that these components only accounted for variation right after the pulse (100-200 ms) over occipital areas (i.e. under the site of stimulation). These components were removed from this participants EEG-data, in addition to components related to eye-movements.*
