# Supplementary material for: Behavioral and electrophysiological evidence for fast emergence of visual consciousness
Source: Neurosci Conscious. 2015 Jul 30;2015(1):niv004. doi: 10.1093/nc/niv004 (PMC6368270; doi:10.1093/nc/niv004)
Supplement: Supplementary Data [file niv004_Supplementary_Data.zip › Supplementary_Figure_2.docx]

*
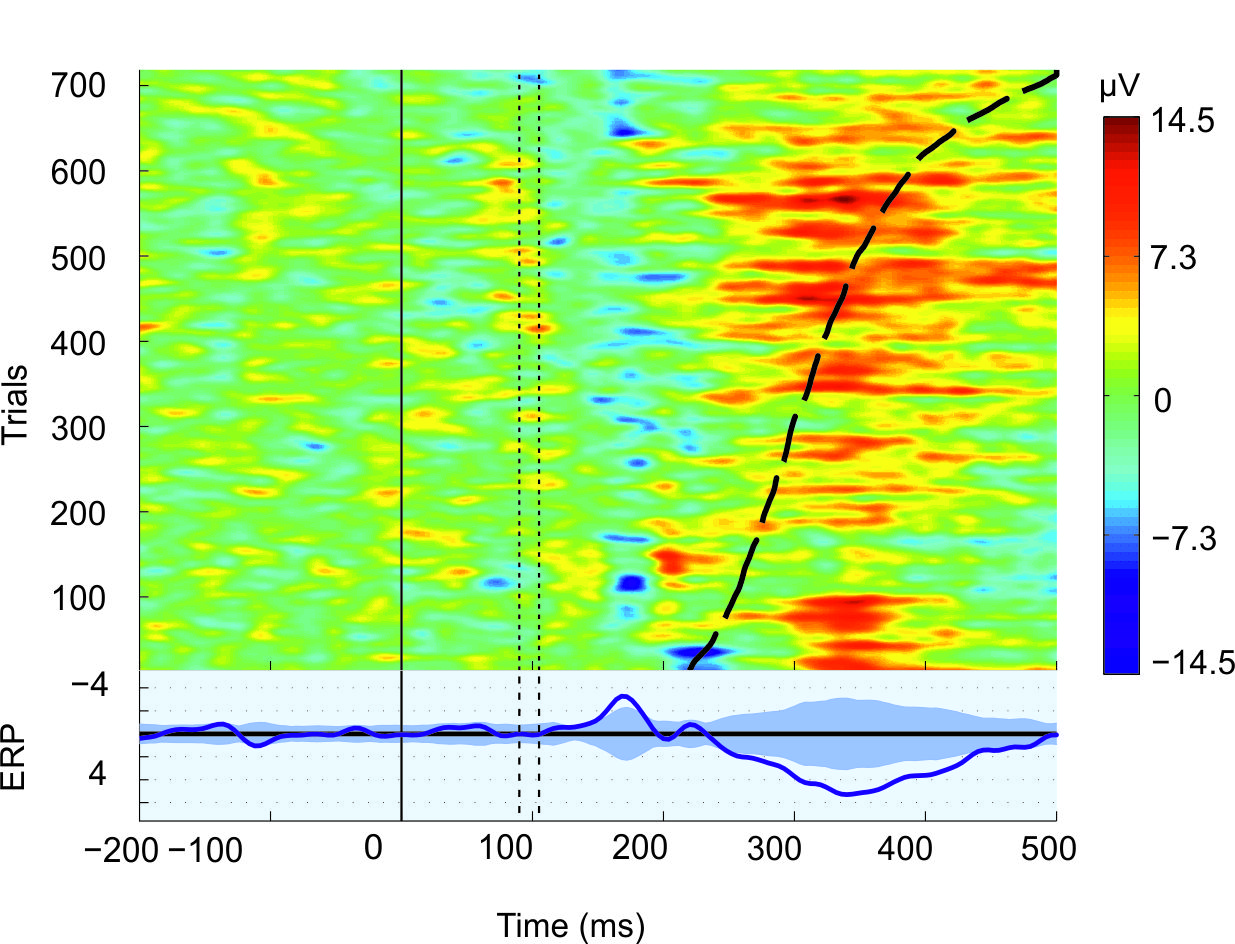
*

*Supplementary Figure 2. ERP-image of the same single-trial differences as in Figure 5, but without de-noising (6 participants, parietal channel Pz). The same NCCs (i.e. VAN and LP) are visible, but the data also includes considerable amounts of noise, especially in the baseline and right before VAN and LP. As in Figure 5, the trials are sorted by RT (dashed line), and the ERP-image has been smoothed for better visualization (Gaussian smoothing, SD = 4 trials). In the average difference ERP presented in the lower part of the figure, the shaded area represents two-tailed alpha level 0.001 (permutation test). Both VAN and LP reach statistical significance even without the de-noising. The dotted lines show the time-period of the removed TMS-pulse artifact.*
